# Specificity of Human Sulfiredoxin for Reductant and Peroxiredoxin Oligomeric State

**DOI:** 10.3390/antiox10060946

**Published:** 2021-06-11

**Authors:** Tom E. Forshaw, Julie A. Reisz, Kimberly J. Nelson, Rajesh Gumpena, J. Reed Lawson, Thomas J. Jönsson, Hanzhi Wu, Jill E. Clodfelter, Lynnette C. Johnson, Cristina M. Furdui, W. Todd Lowther

**Affiliations:** 1 Department of Internal Medicine, Section on Molecular Medicine, Wake Forest School of Medicine, Medical Center Blvd., Winston-Salem, NC 27157, USA; tforshaw@wakehealth.edu (T.E.F.); julie.haines@cuanschutz.edu (J.A.R.); hwu@wakehealth.edu (H.W.); 2 Center for Structural Biology, Department of Biochemistry, Wake Forest School of Medicine, Medical Center Blvd., Winston-Salem, NC 27157, USA; kinelson@wakehealth.edu (K.J.N.); rgumpena@wakehealth.edu (R.G.); lawsjr18@wfu.edu (J.R.L.); thomasjonsson@yahoo.com (T.J.J.); jeclodfe@wakehealth.edu (J.E.C.); ncst8wolfe@gmail.com (L.C.J.); 3 Center for Redox Biology and Medicine, Wake Forest School of Medicine, Medical Center Blvd., Winston-Salem, NC 27157, USA; 4 Comprehensive Cancer Center, Wake Forest School of Medicine, Medical Center Blvd., Winston-Salem, NC 27157, USA

**Keywords:** redox, peroxiredoxin, sulfiredoxin, thiols, hydrogen sulfide, glutathione

## Abstract

Human peroxiredoxins (Prx) are a family of antioxidant enzymes involved in a myriad of cellular functions and diseases. During the reaction with peroxides (e.g., H_2_O_2_), the typical 2-Cys Prxs change oligomeric structure between higher order (do)decamers and disulfide-linked dimers, with the hyperoxidized inactive state (-SO_2_H) favoring the multimeric structure of the reduced enzyme. Here, we present a study on the structural requirements for the repair of hyperoxidized 2-Cys Prxs by human sulfiredoxin (Srx) and the relative efficacy of physiological reductants hydrogen sulfide (H_2_S) and glutathione (GSH) in this reaction. The crystal structure of the toroidal Prx1-Srx complex shows an extended active site interface. The loss of this interface within engineered Prx2 and Prx3 dimers yielded variants more resistant to hyperoxidation and repair by Srx. Finally, we reveal for the first time Prx isoform-dependent use of and potential cooperation between GSH and H_2_S in supporting Srx activity.

## 1. Introduction

Peroxiredoxins (Prxs) are highly conserved and abundant enzymes with well-established function in protection against oxidative damage [1,2,3] and emerging as regulators of metabolism [4,5], signaling [6,7], and DNA stability [8,9,10,11]. Evidence continues to demonstrate key roles in many diseases including Alzheimer’s disease [12,13], diabetes mellitus [14,15], aging [16,17,18,19], cancer [20,21,22], and cardiovascular disease [14,23,24]. Thus, an understanding of the interplay between the activity and structure of Prxs, their chemical mechanisms and intermediates, reducing systems, their inactivation during oxidative stress, and their repair by the enzyme sulfiredoxin (Srx) has the potential to lead to novel therapeutic approaches and biomarkers.

**Figure 1:**
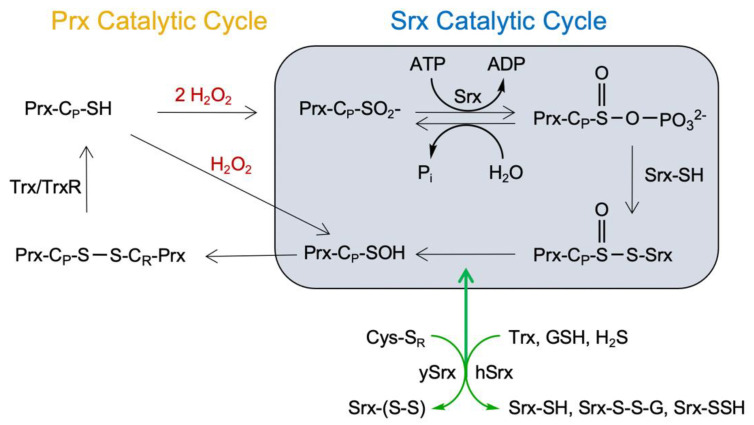
Peroxiredoxin catalytic and sulfiredoxin repair cycles. The resolution of the Prx-Srx complex involves the reduction of the thiosulfiate intermediate (Prx-C_P_-S=O-S-Srx) to yield the Prx Cys-sulfenic acid intermediate (Prx-C_P_-SOH). Yeast Srx contains an adjacent resolving Cys residue (Cys-S_R_) that can react with the thiosulfinate intermediate leading to the formation of an Srx intramolecular disulfide (Srx-(S-S)). In contrast, human Srx has only one Cys residue and requires an exogenous reductant. Possible reductants include the Trx system (Trx/TrxR/NADPH), glutathione (GSH) and hydrogen sulfide (H_2_S); these reductants would ultimately yield reduced Srx (Srx-SH).

The six isoforms of mammalian Prxs are divided into three classes based on mechanistic and structural considerations [1]. The studies presented herein focus on the primarily cytosolic isoforms, Prx1 and Prx2, and the mitochondrial Prx3 isoform. These proteins all belong to the typical 2-Cys family of Prxs (also known as the Prx1 class) with a common mechanism of catalysis involving an intermolecular disulfide bond formation (Figure 1) across an obligate homodimer. However, differences exist in the oligomeric organization of the dimers depending on the redox state. In the reduced state, Prx1 and Prx2 arrange into decameric, toroidal rings that contain five head-to-tail homodimers, while Prx3 forms dodecamers (six head-to-tail homodimers). During catalysis, the *N*-terminal peroxidatic cysteine thiol (C_P_-SH) reacts rapidly with a substrate peroxide molecule (H_2_O_2_ for human Prxs 1–3) and is oxidized to a sulfenic acid (C_P_-SOH). This intermediate then condenses to form a disulfide bond with resolving cysteine thiol (C_R_-SH) on the adjacent Prx monomer of the homodimer, releasing water, and dissociating the multimeric ring structure into homodimers. The extent to which the high order structure collapses differs between these isoforms, with Prx3 having the least stable quaternary structure [25,26]. In cells, the reduction of the disulfide to reduced thiols is predominantly catalyzed by the thioredoxin (Trx)-thioredoxin reductase (TrxR)-NADPH system [1,3]. These molecular transitions have recently been confirmed using an ingenious new in cellulo method [27], which can be mimicked in vitro by the cyclic treatment of 2-Cys Prxs with H_2_O_2_ and dithiothreitol (DTT) [28,29,30].

During formation of the disulfide, a large conformational change from a fully folded (FF) to locally unfolded (LU) active site occurs [2]. The time required for this transition influences whether a second peroxide can react with the C_P_-SOH to further oxidize it to a sulfinic acid (C_P_-SO_2_H), referred to as the hyperoxidized state. When hyperoxidized, Prxs are inactive, enabling buildup of localized H_2_O_2_ and signaling functions. Prx-mediated signaling can occur either through direct H_2_O_2_-mediated oxidation of proteins or thiol-disulfide relay reactions from the disulfide oxidized Prx [31]. Importantly, while the rate of C_P_-SOH formation is similar between Prxs (10^5^–10^8^ M^−1^ s^−1^), unique structural and sequence differences between isoforms dictate sensitivity to hyperoxidation and rate of repair. In other words, the Prx isoforms that form disulfides more quickly are protected from hyperoxidation, suggesting a hierarchy of antioxidant and signaling functions [2,26,29,32,33].

Some of the sequence differences of importance for the sensitivity to H_2_O_2_-induced hyperoxidation of Prxs are located within the C-terminus near the C_R_-SH residue and the YF motif [29,34]. For example, Prx3 has four unique amino acids (Asn232, Thr234, Asp236, and Pro238) in positions otherwise conserved in the other 2-Cys Prx isoforms. Mass spectrometry (MS)-based kinetic studies of chimeras between Prx2 and Prx3 in this region (referred to as CT*) revealed a significant contribution to modulating sensitivity to hyperoxidation. Specifically, exchanging the CT* region in Prx2 with the corresponding region from Prx3 led to a Prx3-like resistant enzyme, while the introduction of the corresponding Prx2 amino acids in Prx3 increased Prx3 sensitivity to hyperoxidation [26,29]. These studies also evaluated a region near the conserved GGLG motif [2]. Chimeras between Prx2 and Prx3 in this region (referred to as PP/HA or HA/PP, respectively, representing the His (H), Ala (A), and Pro (P) residues swapped between the isoforms) had a more subtle effect on the sensitivity to hyperoxidation.

An equally important cellular process is the repair and reactivation of the hyperoxidized Prxs by Srx [31,35,36]. Importantly, the C-terminus of Prxs and the GGLG motif region also establish key interactions with Srx [36,37], and it is currently unknown how the sequence differences in these regions influence the repair reaction. Other key open questions for the field addressed herein regard the preferred oligomeric state of hyperoxidized Prxs for the Srx reaction, and the isoform-specific nature of the reductants used by human Srx. The latter issue is important because human Srx does not have a second Cys residue that can resolve or reduce the Prx-Srx thiosulfinate intermediate (Figure 1), and therefore must rely upon an exogenous reductant. In contrast yeast Srx does possess a resolving Cys [38].

The data presented here show that Prx repair occurs much more efficiently with the Prx decamer/dodecamer than with the dissociated homodimers. Moreover, swapping key amino acids in the Prx2 and Prx3 proteins, changed both their sensitivity to hyperoxidation and ease of repair by Srx, demonstrating the finely tuned structural co-regulation of the two activities. Experiments using different reductants in the reaction also support a preference for H_2_S as reductant for the repair of mitochondrial Prx3, and cooperation between GSH and H_2_S to achieve the most effective repair of the primarily cytosolic Prx2. These findings are important to understand unique mechanisms controlling the activity of Prx isoforms.

## 2. Materials and Methods

### 2.1. Protein Preparation

Wild-type human Srx, Prx1, Prx2, Prx3, and Prx2/3 chimeras were expressed and purified as described previously: Prx2 (WT; PP/HA (P98H and P102A); CT* (G175N, K177T, G179D, and D181P; and PP/HA+CT*) and Prx3 (WT; HA/PP (H155P and A159P); CT* (N232G, T234K, D236G, and P238D; and HA/PP+CT*) [29,32,36,37]. Importantly, all of the proteins used did not contain any remaining affinity tags that have been shown to alter reactivity and oligomeric state [34]. The Prxs were also stored in the disulfide-bonded form to prevent hyperoxidation during storage. The engineered dimer variants Prx2-T82E and Prx3-S139E/A142E, were expressed as an *N*-terminal His-tag fusion using the pET19pp and pET15b vectors (Invitrogen), respectively in BL21Gold (DE3) *E. coli* cells and purified using HisPur Cobalt affinity (Thermo Scientific). The Histag of these proteins was removed with HRV-3C protease (Prx2-T82E; Prx2-E) or thrombin (Prx3-S139E/A142E; Prx3-EE), and the protein was further purified with Q-Sepharose anion exchange and preparative Superdex 200 size exclusion columns. The disulfide-bonded complex between Prx1 and Srx used for crystallization was generated by treating the purified Prx1 variant (C71S, C83V and C173S) with 1 mM 5,5′-dithiobis-(2-nitrobenzoic acid) (DTNB). Excess DTNB and generated TNB^2+^ were removed from the TNB-linked Prx1 using the size exclusion column. Srx was titrated into the Prx1 solution until no further release of TNB^2+^ was observed at 412 nm (~1.5-fold excess Srx over Prx1). The resulting disulfide-bonded Srx-Prx1 complex (i.e., Cys99 of Srx disulfide bonded to Cys52 of Prx1) was passed over the Superdex 200 column to remove excess Srx and TNB^2+^. The complex was concentrated, aliquoted, flash frozen with liquid nitrogen, and stored at −80 °C. All site-directed Srx mutants were generated using the QuikChange Site-directed Mutagenesis Kit from Stratagene. All Srx variants were analyzed by circular dichroism spectroscopy (JASCO-720) in 20 mM HEPES pH 7.5, 100 mM NaCl at a concentration from 0.4 mg mL^−1^ and confirmed to exhibit WT-like spectra.

### 2.2. Crystallization and Structure Determination

Crystals of the Srx-Prx1 complex were obtained by hanging-drop vapor diffusion. Equal volumes of protein (10 mg mL^−1^ in 20 mM HEPES pH 7.5, 100 mM NaCl) and well solution (100 mM citric acid pH 4.5, 26% PEG 400, 100 mM CsCl) were mixed. Crystals were mounted in nylon loops and cryo-cooled at −170 °C for data collection. A single wavelength (1.1 Å) dataset was collected on beamline X29 at NSLS, Brookhaven National Laboratory. Diffraction intensities were integrated using HKL3000 and scaled to 3.0 Å [39]. Cross-validation was performed with 5.0% of the reflections that were set aside. The space group of the crystal was *C*2 with unit cell dimensions *a* = 330.8 Å, *b* = 109.9 Å, and *c* = 260.1 Å and *α* = 90°, *β* = 122.3°, and *γ* = 90°. The structure was solved by molecular replacement using PHASER [40]. A search model was generated by superimposing five dimeric Srx-Prx1 complexes (PDB entry 2RII) onto the five Prx dimers within the decameric Prx2-SO_2_H (PDB entry 1QMV) structure [36,41]. The entire active site helix of Prx1 (residues 46–69) and the C terminus (residues 169–199) were removed from the search model to reduce bias. Two decamers, each containing ten Prx and ten Srx molecules, were found in the asymmetric unit, consistent with the observed self-rotation function. The molecular replacement solution was first refined with rigid-body refinement using REFMAC5 [42]. Iterative rounds of model building with COOT and refinement with local NCS restraints produced R_work_ and R_free_ of 19.2% and 23.0%, respectively, using all diffraction data [43]. The structure was validated using the MOLPROBITY server [44]. Ramachandran plots were prepared with PROCHECK [45]. The data collection and refinement statistics for the structure are summarized in Table S1. Intermolecular surface accessible areas were calculated using AREAIMOL within the CCP4 package [46]. A phosphate ion was observed in the Srx active site in 17/20 molecules; one active site was empty; and 2 contained additional density that was modeled as the triphosphate. All structural illustrations were generated using PyMOL (Schrödinger).

### 2.3. Preparative Scale Hyperoxidation

Preparation of hyperoxidized Prx2 and Prx3 variants was performed at 1 mg/mL (46 µM) Prx in 20 mM HEPES pH 7.5, 100 mM NaCl with volumes ranging from 0.1 to 50 mL. Prx solutions were treated with DTT (50 mM) for 5 min, and H_2_O_2_ was added to a final concentration of either 0.2 mM (Prx2 WT and mutants), 2.0 mM (Prx3 WT and tail mutants), or 6 mM (Prx3-EE mutant). The resulting solution was incubated for 30 min at 30 °C with mixing at 100 rpm. A 40 μL aliquot was removed at different time points, desalted using a Bio-Gel P6 spin column (Bio-Rad) pre-equilibrated with 0.1% formic acid, and analyzed by Electrospray Ionization Time-of-Flight mass spectrometry (ESI-TOF MS) to monitor the oxidation of Prx-C_P_-SH to Prx-C_P_-SO_2_H. Additional H_2_O_2_ aliquots were added as needed to continue Prx cycling until most of the Prx-C_P_-SH was consumed while minimizing the amount of Prx-C_P_-SO_3_H formed.

### 2.4. SEC-MALS Analysis to Measure Prx Size Distribution

Approximate molecular weights of Prx proteins were determined using size exclusion chromatography coupled with multiangle light scattering (SEC-MALS) analysis. Prx proteins were diluted to 2 mg/mL (86–93 µM) in SEC-MALS buffer (50 mM HEPES, pH 7.5, 100 mM NaCl, HPLC-grade water) to a final volume of 0.12 mL prior to resolution on a TSK Gel 4000 SW gel filtration column (8 μm particle size, 7.8 × 30 cm, Tosoh Biosciences). Reduced samples included 10 mM DTT and oxidized samples included 1.2 equivalents of H_2_O_2_. Chromatographic separation was performed on 100 µL (0.2 µg) Prx protein over 35 min at a flow rate of 0.5 mL/min, monitoring elution spectrophotometrically at 280 nm and MALS using a HELIOS II detector (Wyatt Technology). Molecular weights were estimated for prominent peaks (determined from the OD_280_ traces) from MALS data using Astra version 6 software (Wyatt Technology, Santa Barbara, CA, USA).

### 2.5. HPLC Srx Activity and Binding Assays

Srx activity and fluorescence polarization binding assays with hyperoxidized Prx variants were performed as previously described [36]. Briefly, 50 μM hyperoxidized Prx1, 10 μM WT Srx or variant, 50 mM Tris pH 7.5, 100 mM KCl, 1 mM ATP, 1 mM MgCl_2_, and 2 mM DTT were incubated in a 30 µL reaction and stopped at various times by the addition of 15 μL 1 M H_3_PO_4_. Five µL of the sample was injected onto a Waters analytical HPLC system. Different species were separated on a C4 column (Vydac) using a 60–63% acetonitrile/0.1% TFA gradient over 19 min. The fraction of hyperoxidized Prx1 is reported as the mean ± S.D based on peak areas. For the binding studies MgCl_2_ was omitted to prevent turnover, and the binding curves were fit to a single site, saturable model using SigmaPlot.

### 2.6. HRP Assay to Measure Peroxidase Activity

The rate of sulfenic acid formation was determined using the HRP competition assay as described [47]. All assays were performed in triplicate or quadruplicate and contained 15 µM HRP, and 3 µM hydrogen peroxide and varying concentrations of either wild-type Prx2 (0, 2, 4, 8, 12 or 16 µM), Prx2 T83E (Prx2-E; 0, 8, 12, 16, 10, or 24 µM), wild-type Prx3 (0, 2, 4, 6, 8 or 12 µM), or Prx3 S139E/A142E (Prx3-EE; 0, 8, 12, 16, 10, or 24 µM). Four to six independent replicates were performed for each protein and significance was analyzed by two-way ANOVA with a Tukey HSD post hoc correction using Prism version 7.04 (GraphPad, San Deigo, CA, USA).

### 2.7. ESI-TOF MS Srx Activity Assay

Reductive repair of Prx-C_P_-SO_2_H to Prx-C_P_-SH by Srx was carried out in a thermomixer (Eppendorf) at 37 °C with mixing at 500 rpm in a reaction containing 50 μM Prx, 10 μM Srx, 1 mM ATP, 1 mM MgCl_2_, and one or more reducing agents (DTT, GSH, or Na_2_S/H_2_S) at 1–4 mM. Two components of the reaction mixture were prepared separately: one contained Prx, ATP, MgCl_2_ in 20 mM HEPES pH 7.5, 100 mM NaCl, and the other contained Srx and the electron donor(s) (DTT, GSH, H_2_S) in the same HEPES/NaCl buffer. Temperature pre-incubation proceeded for 5 min and then the solutions were mixed. For analysis, 40 μL aliquots were removed at various timepoints and desalted with Bio-Gel P6 spin columns pre-equilibrated with 0.1% formic acid in water to quench the reaction. Columns were centrifuged for 4 min at 1000× *g*, and the eluate was analyzed via direct infusion using ESI-TOF MS. Time zero spectra, revealing the initial amount of Prx redox species, were acquired either by treatment of Prx (50 μM) with DTT (2 mM) in the HEPES/NaCl buffer or by incubation of a complete reaction minus ATP for 10 min at 37 °C, followed by desalting and MS analysis. Reactions were performed in triplicate or duplicate, with each replicate on either separate days or in parallel on the same day in separate tubes. ESI-TOF MS analyses were performed on an Agilent 6120 MSD-TOF system operating in positive ion mode with the following settings: capillary voltage of 3.5 kV, nebulizer gas pressure of 30 psig, drying gas flow of 5 L/min, fragmentor voltage of 175 V, skimmer voltage of 65 V, and dry gas temperature of 325 °C. Samples were introduced via direct infusion at a flow rate of 20 μL/min using a syringe pump (KD Scientific). Mass spectra were acquired over the range of 500–3200 *m*/*z*, then averaged, deconvoluted, and ion abundance quantified using MassHunter Workstation software version B.02.00 (Agilent, Santa Barbara, CA, USA. Relative ion abundances were used to determine the amounts of each relevant species at a given time point. Reaction rates were determined by fitting to the appropriate equations in Prism version 7.04 (GraphPad, San Deigo, CA, USA). Two to four independent replicates were performed for each protein and significance was determined by multiple unpaired two-tailed *t*-tests using GraphPad Prism version 7.04.

## 3. Results

### 3.1. Decameric Srx-Prx1 Complex Reveals Extended Binding Interface

Structural and biochemical studies have shown that human Prx1 and Prx2 form a decameric toroid. In our previous studies to understand the structural basis for the ability of Srx to reduce the hyperoxidized form of human Prxs, we relied upon an engineered Prx1 dimer (C83E) in order to obtain crystals of diffraction quality [36,37]. These studies enabled us to understand the juxtaposition of the two active-site interfaces of the two proteins and the wrapping of the Prx C-terminus around Srx in an essential interaction that we called the “embrace” [36]. However, it is still not clear whether or not the Srx-Prx interactions are the same when the Prx is present in the higher order oligomeric state, and which oligomeric state of the Prx is the preferred substrate for Srx.

In order to determine the full decameric Prx-Srx complex, we generated the Prx1 C83V mutant, selected based on previous studies showing that the decamer of the homologous variant in the bacterial AhpC peroxiredoxin was stabilized [48]. The most important part of this strategy was to convert all of the remaining Cys residues to Ser in Prx1 molecule (C71S, C173S) except for the C_P_ residue (Cys52), and then to treat this Prx1 variant with DTNB (Ellman’s reagent) to generate a mixed disulfide at Cys52 (C_P_-S-TNB) [36,37]. The TNB-modified Prx1 was then titrated with Srx until 100% of the disulfide linked Prx-Srx complex was formed. This process was necessary to produce a homogenous, fully complexed sample and high-quality crystals.

**Figure 2:**
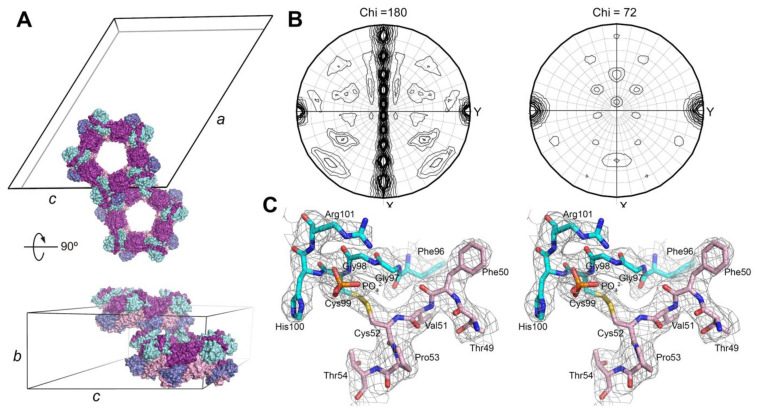
Crystal structure of the Srx complex with the Prx1 decamer. (**A**) Packing of the complex within the *C*2 unit cell. Two toroid-shaped complexes containing ten Prx dimers (violet/purple) and twenty Srx molecules (cyan/blue) were found in the asymmetric unit. Cell dimensions: *a* = 330.8 Å, *b* = 110.0 Å, *c* = 260.2 Å, *α* = 90°, *β* = 122.3°, *γ* = 90°. (**B**) Self-rotation function calculated using the program MOLREP with data in the 10–4 Å resolution range and an integration radius of 30 Å. The χ = 180° plot contains ten two-folds separated by 18°. These axes are perpendicular to a five-fold axis indicated on the χ = 72° plot, consistent with the Srx-Prx complex having 52-point group symmetry. (**C**) Stereoview of representative 2*F*_o_–*F*_c_ electron density map centered on the engineered Srx-Prx disulfide contoured at 1 σ.

The Srx-Prx1 complex diffracted to 3.0 Å resolution (Supplementary Table S1) and was solved by molecular replacement. Two 341 kDa toroid-shaped decamers, each composed of five Prx dimers and ten Srx monomers, were within the asymmetric unit (Figure 2A). The presence of ten two-fold axes (Chi = 180) (Figure 2B) in the self-rotation function, perpendicular to a five-fold symmetry axis (Chi = 72) and coincident with the *b* cell edge direction, confirmed the cell packing. The 2*F*_o_−*F*_c_ electron density map confirmed the presence of the engineered disulfide bond between Cys52 of Prx1 and Cys99 of Srx, a mimic of the thiosulfinate reaction intermediate, and a phosphate molecule within the ATP binding pocket of Srx (Figure 2C) [36,37].

**Figure 3:**
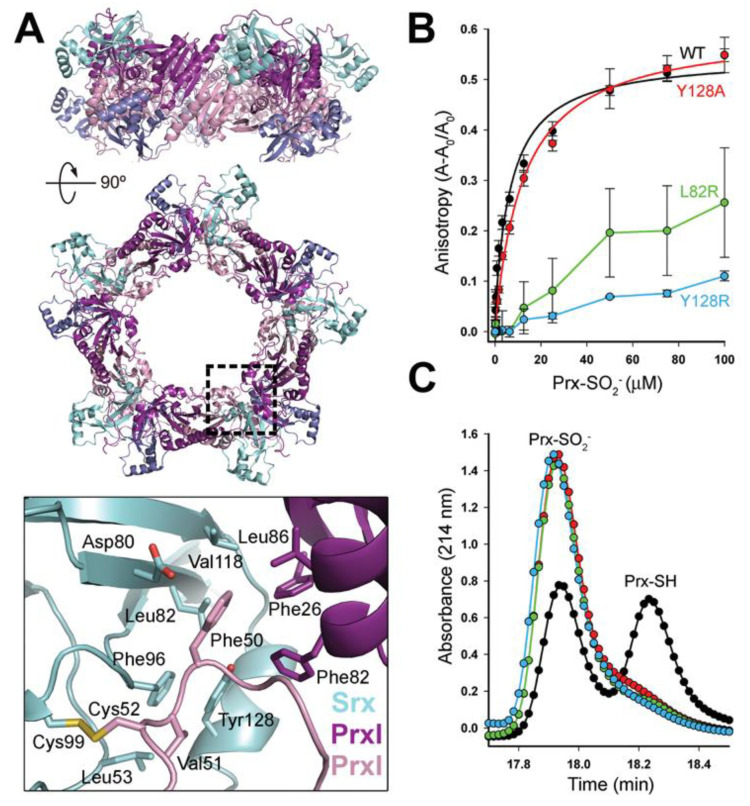
Adjacent Prx molecule at the dimer-dimer interface contributes to the Prx-binding pocket of Srx. (**A**) Cartoon representation of the Srx-Prx1 complex in orthogonal views; same coloring used as in Figure 2. The lower panel shows details of the dimer–dimer interface proximal to the Srx active site, indicated by the dashed box in the upper panel. Phe50 of Prx1 (violet) interacts with a hydrophobic pocket (cyan) formed by Leu53, Asp80, Leu82, Phe96, Val118, and Tyr128 of Srx. The Prx1 subunit (purple) from a neighboring dimer in the decamer contributes Phe26, Phe82 and Leu86. (**B**) Biochemical analysis of Srx variants within the Phe50 binding pocket. Srx interactions measured in solution by changes in fluorescence anisotropy with Oregon Green 514-labelled Srx variants. The data are expressed as the fractional change in anisotropy, (A − A_0_)/A_0_, versus the concentration of decameric Prx1-SO_2_H added, with the error bars indicating s.d. (**C**), Representative HPLC traces from the activity analysis of Srx variants; 30 min time point shown. Repair of the Prx1-SO_2_H moiety by WT Srx leads to a marked shift to a later elution time.

The structure of the dimeric building unit of the Srx complex with the Prx1 decamer was similar to the structure with the Prx1 dimer previously solved, with the Srx molecules sandwiched between the active site surface of one Prx1 monomer and the C-terminal tail from the adjacent Prx1 monomer [36]. This part of the complex resulted in the burial of 700 Å^2^ at each Srx-Prx active site interface and 900 Å^2^ between the C-terminal tail and the backside of Srx monomer (Figure 2A). Importantly, the new structure also revealed that the Prx1 subunit from a neighboring dimer provides additional interactions with both the Prx monomer to be repaired (pink in Figure 3A; 725 Å^2^) and its associated Srx molecule (cyan; 418 Å^2^). Phe26, Phe82, and Leu86 of the adjacent Prx1 molecule (purple in Figure 3A) essentially forming the second half of the Phe50 binding pocket. The remaining residues of this predominantly hydrophobic pocket come from Srx (Leu82, Phe96, Val118, Tyr128).

### 3.2. Dimer-Dimer Interface Disruption Decreases Srx Binding and Repair of Prx1

In the Srx repair mechanism, it is essential for the buried Cys sulfinic acid-containing helix of Prx1 to locally unfold and to move ~10 Å to approach the ATP molecule bound within the Srx active site [37]. The observation that the binding pocket for Prx1 Phe50 is made up of residues from Srx and the adjacent Prx1 subunit (Figure 3A) suggests that the absence of these interactions will prevent Srx-mediated repair. To test this hypothesis, Leu82 or Tyr128 in Srx were mutated to Arg to repel the Prx1 Phe50 loop binding. The Tyr128Ala Srx variant was also made to test whether making the surface pocket larger would alter the ability of the Prx1 Phe50 loop to bind to Srx.

The ability of the Srx variants to bind wild-type (WT), decameric Prx1-SO_2_H was tested by fluorescence anisotropy (Figure 3B) using Oregon Green labeled Srx [36]. The binding constant of WT Srx for Prx1 was determined to be 7.0 ± 1.5 µM, consistent with our previously determined value [36]. In contrast, the Leu82Arg and Tyr128Arg variants of Srx had significantly reduced or no binding. Removal of the *p*-hydroxyphenyl sidechain in the Tyr128Ala mutant resulted in a binding constant of 12.5 ± 1.2 µM, similar to WT Srx. The catalytic activity of the Srx mutants was also monitored by HPLC chromatography, using our previous protocol [36] (Figure 3C). The Leu82Arg and Tyr128Arg Srx mutants both exhibited no activity. These observations indicate that the introduction of a charged residue in the hydrophobic Prx1 Phe50 binding cavity decreased binding of Srx to Prx1 and abolished repair activity. Surprisingly, the Tyr128Ala Srx mutant, which preserves some of the hydrophobicity within the cavity, binds to Prx1 with WT-like properties, but only showed activity when incubated at equimolar concentration of the Prx1 (data not shown). Taken together these observations illustrate the delicate nature of the hydrophobic interfaces formed between Srx and two Prx1 molecules at the Prx dimer-dimer interface, in order to correctly position Phe50 and the C_P_-SO_2_H moiety for reduction by Srx.

### 3.3. Higher Order Oligomeric State of Prx2 and Prx3 Is Also Required by Srx

While the experiments in the previous sections strongly support the necessity of the decamer oligomeric state for the repair of human Prx1 by Srx, we sought to test whether this was also true for Prx2 and Prx3. For these experiments, MS-based kinetic analysis was employed to monitor the progress of the reaction using ESI-TOF MS, as done previously [29,36]. We chose to focus at this point on Prx2 and Prx3, as we had the opportunity to compare the results to our previous studies that used a panel of chimeras and other mutants of these proteins [26,29,32]. Another advantage of this approach is that it allows one to monitor the repair reaction even for Prx variants that are inefficiently hyperoxidized, such as Prx3, and to interrogate complex mixtures of Prx species.

**Figure 4:**
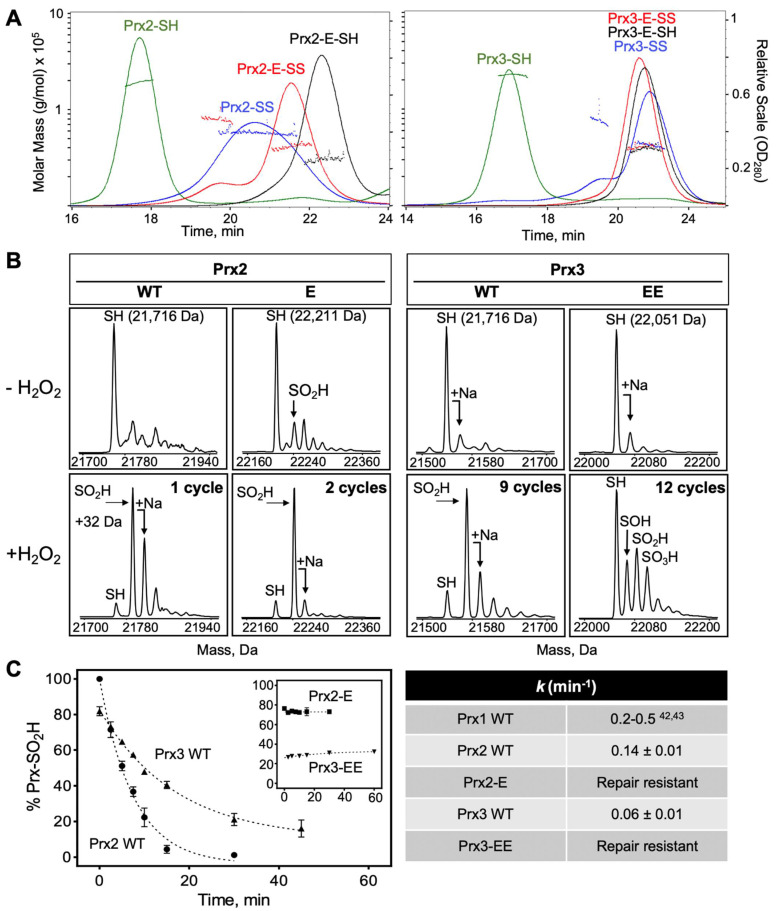
Comparison of the structural and redox properties of Prx2 and Prx3 variants. (**A**) SEC-MALS analysis of WT and engineered dimers of Prx2 and Prx3 in different oxidation states. *Left*, Prx2 variants *Right*, Prx3 variants. (**B**) Hyperoxidation of Srx-mediated repair for Prx2 and Prx3 WT and variants as monitored by ESI-TOF MS. Hyperoxidation of Prx2 (left series) and Prx3 (right series) carried out by addition of H_2_O_2_ (Prx2 WT and -E mutant 0.2 mM H_2_O_2_; Prx3 WT 2.0 mM H_2_O_2_, Prx3-EE 6 mM H_2_O_2_) every 30 min (one cycle) to 1 mg/mL Prx in the presence of DTT (50 mM). Protein spectra are shown prior to H_2_O_2_ addition (top) and subsequent hyperoxidation with the indicated number of cycles (bottom). (**C**) Time course analysis of Prx-SO_2_H reduction catalyzed by Srx in the presence of DTT. The reaction progress was assessed at the indicated time points, and the data represent Prx-SO_2_H signal abundance as a percent of the total ion abundance accounting for all prominent species in the mass spectra (*n* = 3, Prx3-EE *n* = 2). The data for the WT proteins were fit to an exponential decay equation to compare the relative reaction rates for the repair.

To test the hypothesis that the dimer–dimer Prx interface within the higher oligomeric state contributes positively to the repair by Srx, WT Prx2, and Prx3 and engineered dimers of each protein were expressed and purified to homogeneity. This question is of particular interest for Prx3 since the dimer–dimer interface within the dodecamer is different from that for decameric Prx1 and Prx2 [34,49]. For Prx2 we have found that the single Thr82Glu mutation (denoted in the figures as Prx2-E for simplicity) was sufficient to maintain the protein in the dimeric state at high concentration (>25 mg/mL) and independent of oxidation state (Figure 4A, left panel), as monitored by size-exclusion chromatography with multi-angle light scattering (SEC-MALS). This observation is consistent with all of our previous work using the analogous Prx1-E (Cys83Glu) variant [36]. In contrast, Prx3 required two mutations (Ser139Glu and Ala142Glu; denoted Prx3-EE) to maintain the dimeric state (Figure 4A, right panel), when present in either reduced or oxidized states. This method also clearly demonstrates that the WT Prx3 dodecamer (~16.7 min) elutes earlier that the decamer of Prx2 (~17.5 min). All Prx2 and Prx3 variants exhibit comparable rates of reaction with H_2_O_2_ (*k*_SOH_ = 0.9 − 3 × 10^7^ M^−1^ s^−1^) using the HRP competition assay (Supplementary Table S2), supporting that the dimeric variants were not enzymatically compromised [47]. However, as indicated below, their sensitivity to hyperoxidation was dramatically altered.

The Prx2 and Prx3 variants were hyperoxidized on large scale using cycles of H_2_O_2_ and DTT addition as described in the Methods section. As we and others have reported, WT Prx3 is significantly more resistant to hyperoxidation than WT Prx2 (Figure 4B), requiring more time and cycles of H_2_O_2_ and DTT treatment to become hyperoxidized to the C_P_-SO_2_H state (masses observed are consistent with a+32 Da increase) [26,29,32]. The same trend holds for Prx2-E and Prx3-EE. Prx2-E can be hyperoxidized given enough time, but Prx3-EE remains predominantly in the reduced state (C_P_-SH). A small amount of Prx3-EE did form the sulfenic (C_P_-SOH), sulfinic (C_P_-SO_2_H) and sulfonic (C_P_-SO_3_H) acid species.

Hyperoxidized WT Prx2 and Prx3 were repaired by Srx (Figure 4C) with rates comparable (0.14 and 0.06 min^−1^, respectively) to what we and others have previously reported for Prx1 with DTT as reductant (0.14–0.50 min^−1^) [36,50,51]. In contrast, both dimeric Prx2-E (~80% hyperoxidized at the start of the reaction) and Prx3-EE (~20% hyperoxidized) were not repaired or reduced by Srx (Figure 4C inset). Thus, these data support the notion that decameric Prx1 and Prx2 and dodecameric Prx3 are the preferred substrates for human Srx. It is interesting to note here, however, that the repair of Prx3 is slower than that of Prx2 when using DTT as electron donor.

### 3.4. C-Terminal Sequence Differences between Prx2 and Prx3 Impact the Rate of Repair by Srx

The preceding experiments focused on the active site interactions between Srx and Prx and clearly support that the higher order oligomeric state is one key component of the interaction. The other major interface between the two proteins is the backside interface (Figure 2 and Figure 3). We have shown that the complementary interactions between Srx and Prx are required for binding and repair by Srx [36]. Importantly, the C-terminus of the Prx molecule contains the resolving cysteine residue. Using chimeras between Prx2 and Prx3 in this region, we have also shown that these residues influence the kinetics of intermolecular disulfide bond formation and sensitivity to hyperoxidation [26,29].

The question now is whether or not these C-terminal regions also impact the Srx-mediated repair process. Four different constructs were purified and hyperoxidized as before: WT Prx2 and Prx3; C-terminal sequence swap (CT*), a region near the active site GGLG motif (Prx2, PP/HA; Prx3, HA/PP); and the combination (Prx2, PP/HA+CT*; Prx3, HA/PP+CT*) [29]. Supplementary Figure S1 illustrates the starting material for each protein and representative mass spectra at different time points that highlight the differences in sensitivity to hyperoxidation.

**Figure 5:**
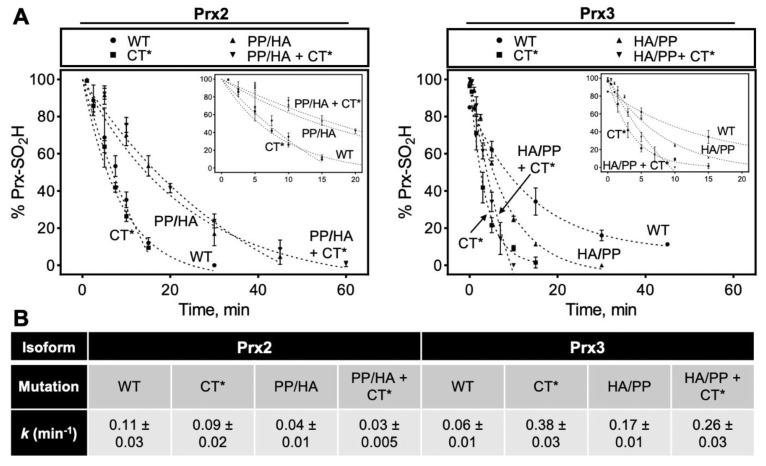
Repair of hyperoxidized Prx2, Prx3 and their chimeras. Time course analysis of Prx-SO_2_H reduction catalyzed by Srx in the presence of DTT. (**A**) The reaction progress was assessed at the indicated time points by ESI-TOF MS analysis and the data represent Prx-SO_2_H signal abundance as a percent of the total ion abundance accounting for all prominent species in the mass spectra (*n* = 3). Inset indicates the first 20 min of the reaction. The data for the WT and chimera proteins were fit to an exponential decay equation to compare the relative reaction rates for the repair of hyperoxidized proteins. (**B**) Summary table of kinetic repair parameters, *k* is shown as ± standard deviation (SD).

The Srx-catalyzed reduction of the hyperoxidized Prx variants was carried out using ATP-Mg^2+^ and DTT as the reductant. The decrease in the C_P_-SO_2_H species over time was measured by ESI-TOF MS and quantified relative to all other species (Figure 5). While the Prx2-CT* variant (0.09 min^−1^) was repaired at essentially the same rate as the WT Prx2 protein (0.11 min^−1^) (WT data from Figure 4C also shown here for comparison), the Prx2 PP/HA and PP/HA+CT* chimera exhibited significantly lower rates (0.03–0.04 min^−1^). In contrast, all of the Prx3 variants (0.17–0.38 min^−1^) showed faster repair than WT Prx3. Interestingly, the Prx3 constructs containing the CT* sequences were reduced more efficiently than WT Prx2. Changes to the GGLG regions for Prx3 appeared to have little effect. In summary, for Prx2 the GGLG region had the highest impact, while for PRX3 the CT region dominated the repair by Srx.

### 3.5. Human Srx Can Utilize GSH and H_2_S as Reducing Source

The strong reducing agent DTT has been used historically to conduct human Srx repair assays in vitro to ensure that the reduction of the thiosulfinate intermediate (Figure 1) involving Cys99 of Srx and the peroxidatic cysteine of Prx was not kinetically limiting. To determine the ability of endogenous small molecule thiol reductants to support the activity of human Srx, we performed side-by-side reactions using GSH and Na_2_S (source of H_2_S, hereafter referred to as H_2_S). We first varied the concentration of these reductants (1–4 mM) to ensure these were not rate limiting for the reaction (data not shown). Based on these experiments, all the repair reactions were performed using 1 mM reductant (DTT, GSH and/or H_2_S).

**Figure 6:**
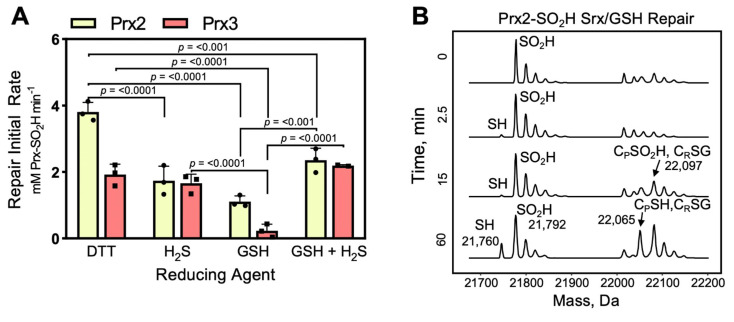
Kinetic study of the repair of hyperoxidized human Prx2 and Prx3 by Srx using chemical and biological reductants. (**A**) Summary bar graph presentation of the rates of Prx-SO_2_H reduction catalyzed by Srx in the presence of DTT, H_2_S, GSH, and combined H_2_S, GSH. Time course data were collected by ESI-TOF MS analysis, and the reaction rates were calculated by fitting the initial decrease in Prx-SO_2_H to a linear equation (*n* = 3). Data shown as ± standard deviation (SD). (**B**) ESI-TOF MS spectra of Prx2-SO_2_H repair by Srx with GSH, showing accumulation of glutathionylated Prx2 over time.

To facilitate direct comparisons, we determined the initial rate of the reaction by fitting the linear portion of the progress curve data within the 10 min time window. Using this approach, DTT supported most efficiently the repair of Prx2 (Figure 6A), and the repair of Prx3 was ~50% lower with DTT. This data supports that the structure of Prx3 influences the formation of the Prx3-Srx thiosulfinate intermediate. Interestingly, the repair of Prx2 was significantly slower when GSH or H_2_S were used as reductants (~2- and 4-fold, respectively). The reaction for Prx3 with H_2_S was similar to the activity with DTT, while the reaction with GSH was the slowest (~10-fold relative to DTT or H_2_S). Since GSH and H_2_S can be simultaneously present in the cytoplasm and mitochondria, we tested their combination (1 mM each). The reduction of hyperoxidized Prx2 was higher than with H_2_S or GSH alone but remained ~2-fold slower relative to DTT. The repair of Prx3 with either H_2_S alone or H_2_S/GSH combination achieved rates comparable with DTT. In summary, Srx-mediated repair of Prx2 can utilize both GSH and H_2_S while Prx3 was more efficient with H_2_S.

To explain the slower Prx2 and Prx3 repair with GSH, we considered the potential of glutathionylated Prx species accumulating during the reaction. This was indeed the case for Prx2 (Figure 6B), where there was notable accumulation of glutathionylated species over the time course of the reaction. Prx3 was also glutathionylated under these conditions but to a much lesser extent (data not shown). Glutathionylation of sulfiredoxin was not observed. Thus, modification of Prx by GSH in vitro affects the ability to efficiently and fully repair the Prx molecules present in solution to a fully reduced state. In cells, however, it is possible these species may be resolved by other reductants (see below) or enzymatically by the glutaredoxin–glutathione reductase system.

Given the findings of Prx2 glutathionylation, we sought to determine if similar accumulation of Prx hydrosulfides would occur in the presence of H_2_S. As the mass of hyperoxidized (C_P_-SO_2_H) and persulfidated Prx2 (C_P_-SSH) are virtually identical in the ESI-TOF MS analysis (+32 Da), an increase in persulfidation will mask the decrease in hyperoxidation during the Srx-catalyzed repair reaction. To resolve this, we performed experiments using Prx2 hyperoxidized with H_2_^18^O_2_, which separates the mass of hyperoxidized and persulfidated protein by 4 mass units. We did not observe the formation of the persulfidated proteins ruling out this hypothesis (data not shown). Another possibility for the slower repair of Prx2 with H_2_S could be the stability of the H_2_S in solution, but this would not be consistent with the almost complete repair of Prx3 in the presence of H_2_S. Thus, the mechanisms responsible for the slower kinetics of Prx2 repair with Srx and H_2_S remain to be elucidated.

## 4. Discussion

The metabolic and signaling implications of Prx enzymes are complex and multilayered. Under low H_2_O_2_ concentrations, multimeric (do)decamer rings of typical 2-Cys Prx enzymes rapidly reduce substrates, are oxidized to disulfide-linked homodimers, which either participate in thiol–disulfide relay reactions to oxidize interacting proteins or are reduced by a Trx-TrxR-NADPH system back to the original multimeric ring structures [1,3]. However, under high H_2_O_2_ conditions, hyperoxidation and inactivation of 2-Cys Prxs results in a change of function to (i) allow H_2_O_2_ to locally accumulate and oxidize less sensitive thiols (floodgate hypothesis) [2], (ii) conserve NADPH as it is no longer utilized to reduce Prx disulfide and thus can be utilized for the Trx-mediated reduction of other oxidized thiols [2], and (iii) gain chaperone or holdase activity [52,53,54]. The repair of the inactivated Prxs by Srx restores peroxidase activity, helping the cell to reestablish the basal redox state. Therefore, an understanding of how human Srx interacts with its substrate and the requirement for exogenous reductants is needed, as the levels of Prx hyperoxidation have been implicated in many disease states.

Previously, we successfully used the engineered, dimeric form of the Srx-Prx1 complex to determine how Srx binds to Prx and positions of the Prx sulfinic acid moiety relative to the ATP molecule, leading to the formation of sulfinic phosphoryl ester and thiolsulfinate intermediates (Figure 1) [36,37]. These studies, however, did not yield structural insights into the repair of Prx toroids. For Prx1, Prx2, and yeast Tsa1, the decamer is stabilized by hyperoxidation in vitro and in vivo [55,56]. For Prx3, the oligomeric state of the hyperoxidized species in cells is not known. Importantly, for the kinetic studies herein, we treated WT Prx3 with H_2_O_2_ until we acquired the fully hyperoxidized, dodecameric species to facilitate and simplify the analysis.

In order to form the Srx-Prx1 decameric complex, we had to stabilize the dimer–dimer interface by installing the C83V mutation. The necessity for doing this may be more related to the crystallization process than what likely occurs in cells, as discussed below. The crystal structure of the decameric Srx-Prx1 complex (Figure 3A) revealed for the first time an intermolecular interface that increases the size of the binding pocket for the partially unfolded Prx active site loop, which contains the Cys sulfinic acid moiety and Phe50. This expanded contact comes from the neighboring Prx dimer, providing an additional ~1200 Å^2^ of buried surface between the Prx being repaired and its associated Srx. Thus, the total contact to Srx encompasses ~2100 Å^2^ from three Prx molecules, which amounts to 28% of the total Srx surface. Mutations in Srx at the interface lead to a loss of Srx binding and repair (Figure 3B,C).

Next, we sought to further investigate the kinetic implications of the newly discovered Prx interface by studying the repair of hyperoxidized Prx2-E and Prx3-EE constructs, which exist exclusively in the dimeric state (Figure 4). These Prx2 and Prx3 variants were significantly more resistant to hyperoxidation than the corresponding WT human proteins, confirming that the packing at the dimer-dimer interface in the multimeric ring structure is also important for reactivity with substrate peroxides (Figure 3A), consistent with studies on the bacterial homolog, AhpC [48]. Importantly, the Srx was unable to catalyze the repair of the dimeric Prx variants.

The inability of Srx to repair dimeric Prx2 and Prx3 has important ramifications for how we think about this process within cells. One possible scenario for Prx1 and Prx2 is that Srx repairs most of the dimers within one assembly simultaneously, leading to a fully reduced active decamer. In an alternative scenario, the association of Srx to multimeric Prx leads to dissociation and repair of Prx dimers, which can then assemble to reform active, reduced decamers. This process is facilitated by the unfolding of the C-terminal region of the Prx and is exemplified by the Srx-Prx backside interface (Figure 3) [36]. The remaining hyperoxidized species could potentially reassemble into a hyperoxidized decamer and enable rounds of Srx-mediated reduction to occur. Perhaps this latter scenario is also part of how Srx dissociates the hyperoxidized, supramolecular chaperone species and why it takes a long time to reduce an entire population of hyperoxidized Prx in vitro and in vivo (Figure 4) [36,57].

Given the difficulty in hyperoxidizing dimeric Prx3 (Figure 4B) and our recent report that the dodecamer of Prx3 readily dissociates predominantly to dimer upon the addition of only two equivalents of H_2_O_2_, it is unclear how a significant population of hyperoxidized Prx3 can occur [25]. Nonetheless, Prx3 hyperoxidation and repair does occur within the mitochondria and is important for the circadian rhythm [58,59]. Additional structural and mechanistic studies are clearly needed to understand Prx3 hyperoxidation and its repair by Srx in the context of the mitochondrial milieu, its reducing systems and available small molecule reductants.

Our studies on the Prx2 and Prx3 chimeras herein demonstrated that the sequences present in the GGLG and C-terminal regions can modulate the efficiency of repair by Srx. Thus, regions of the Prx-Srx interface may have evolved to suit specific signaling needs by controlling the efficiency of Srx-mediated repair. We anticipate these to be revealed from studies of Srx selectivity towards other oxidized protein substrates [60].

One of the unique facets of the Srx reaction is the formation of a thiosulfinate intermediate with the Prx-C_P_ residue [35,36,61]. The mechanism for the reduction of this intermediate varies depending on the number of Cys residues present. For example, yeast Srx contains a second Cys residue that essentially functions like the C_R_ residue of 2-Cys Prxs to collapse the reaction intermediate and to form an intramolecular disulfide bond (Figure 1), which is then reduced by Trx/TrxR system [62]. In contrast, if this second Cys is mutated to Ser, the thiosulfinate intermediate can be reduced by GSH [38]. Human Srx has only the reactive Cys99 and based on analogy to the yeast protein likely relies on either small molecule thiols or thioredoxin to facilitate turnover. Indeed, the repair of human Prx1 by Srx in the presence of GSH and Trx has been demonstrated [50]. However, the function and comparison to other biological thiols such as H_2_S has not been investigated for Prx2 and Prx3.

Thus, we monitored the repair kinetics of hyperoxidized Prx2 and Prx3 by Srx with GSH and H_2_S as reductants relative to the commonly used (though non-biological) reductant, DTT. These initial data demonstrate for the first time that H_2_S can function as an electron donor in the Srx reaction. However, while the repair of Prx3 by H_2_S was approximately as fast as with DTT, the repair of Prx2 was ~2-fold slower with H_2_S than DTT (Figure 6). We ruled out a potential overlap of C_P_-SO_2_H and C_P_-SSH species in MS analysis using isotope labeled H_2_^18^O_2_ to hyperoxidize Prx2. We also eliminated the possibility of insufficient H_2_S concentration as the repair of Prx3 was complete under the same experimental conditions. One remaining possibility is that Prx2 repair by H_2_S is more complex requiring additional components. The repair of hyperoxidized Prx3 is especially intriguing given its mitochondrial localization and the implications for the regulation of mitochondrial redox metabolism. Moreover, the H_2_S generating enzymes 3-mercaptopyruvate sulfurtransferase and cystathionine β-synthase are also transported into the mitochondria upon mitochondrial oxidative signals resulting in increased mitochondrial H_2_S [63,64]. The kinetic data presented herein support the function of H_2_S as a reductant that can support the repair of hyperoxidized Prx3 by Srx in the absence of other reducing systems.

Interestingly, overall GSH was less effective in supporting the Srx-catalyzed repair of Prx2 and Prx3 relative to DTT or H_2_S (Figure 6). There was also a significant amount of glutathionylated Prx detected, in particular for Prx2, indicating incomplete reaction and the need of glutaredoxin (Grx) or other systems to deglutathionylate the proteins and regenerate the reduced Prx species. Combined addition of GSH and H_2_S was able to increase the kinetics of repair for Prx2 relative to H_2_S or GSH, suggesting potential cooperation of the two reducing metabolites in biological settings. Thus, in the absence of an enzymatic system to reduce disulfide or thiosulfinate linked Srx-Prx dimers, the studies herein suggest the possibility of a preference for H_2_S to support the repair of mitochondrial hyperoxidized Prx3 by Srx, and of combined GSH and H_2_S for the repair of cytosolic Prx2. Importantly, future detailed studies will be required to investigate the concentration and time dependence of reductants in vitro, to tease apart the contributions of these reductants in cells, and to investigate the interplay between the GSH recycling system, the appropriate Trx/TrxR system, and the enzymes that produce H_2_S. Based on the recent study by Benchoam et al., glutathione hydropersulfide (GS-SH) should also be added to the list of potential reductants, as its acidity and nucleophilicity is much greater that GSH [65].

## 5. Conclusions

In summary, these studies provide critical structural and biochemical data on the Prx-Srx interaction relevant for the typical 2-Cys Prxs and identify kinetic consequences of quaternary Prx structure, dimer versus (do)decamer, on the sensitivity to hyperoxidation and efficiency of repair by Srx. Moreover, there appears to be different preferences for reducing molecules including GSH and H_2_S. Critically, this work further highlights the phenomenon that despite sharing similar mechanisms of action, sequence homology, and substrate preferences, each peroxiredoxin isoform has a unique signaling role which may in part be driven by differences in the resolution of inactive hyperoxidized state.

## Data Availability

The coordinates and experimental data for the reported, decameric Prx1-Srx structure are available at the Protein Data Bank (7LJ1).

## Supplementary Materials

The following are available online at https://www.mdpi.com/article/10.3390/antiox10060946/s1, Figure S1: Hyperoxidation of Prx2 and Prx3 chimeras monitored by ESI-TOF MS, Table S1: Crystallographic data and refinement statistics, Table S2: *k*_SOH_ formation rates as determined by HRP competition assay.

## References

[1] Hall A., Nelson K., Poole L., Karplus P.A. (2011). Structure-based insights into the catalytic power and conformational dexterity of peroxiredoxins. Antioxid. Redox Signal..

[2] Wood Z.A., Poole L.B., Karplus P.A. (2003). Peroxiredoxin evolution and the regulation of hydrogen peroxide signaling. Science.

[3] Wood Z., Schröder E., Harris J.R., Poole L.B. (2003). Structure, mechanism and regulation of peroxiredoxins. Trends Biochem. Sci..

[4] Kil I.S., Lee S.K., Ryu K.W., Woo H.A., Hu M.-C., Bae S.H., Rhee S.G. (2012). Feedback control of adrenal steroidogenesis via H_2_O_2_-dependent, reversible inactivation of peroxiredoxin III in mitochondria. Mol. Cell.

[5] Woo H.A., Yim S.H., Shin D.H., Kang D., Yu D.-Y., Rhee S.G. (2010). Inactivation of peroxiredoxin I by phosphorylation allows localized H_2_O_2_ accumulation for cell signaling. Cell.

[6] Hopkins B.L., Nadler M., Skoko J.J., Bertomeu T., Pelosi A., Shafaei P.M., Levine K., Schempf A., Pennarun B., Yang B. (2018). A peroxidase peroxiredoxin 1-specific redox regulation of the novel FOXO3 microRNA target let-7. Antioxid. Redox Signal..

[7] Stöcker S., Maurer M., Ruppert T., Dick T.P. (2018). A role for 2-Cys peroxiredoxins in facilitating cytosolic protein thiol oxidation. Nat. Chem. Biol..

[8] Aeby E., Ahmed W., Redon S., Simanis V., Lingner J. (2016). Peroxiredoxin 1 protects telomeres from oxidative damage and preserves telomeric DNA for extension by telomerase. Cell Rep..

[9] Ahmed W., Lingner J. (2018). PRDX1 and MTH1 cooperate to prevent ROS-mediated inhibition of telomerase. Genes Dev..

[10] Ahmed W., Lingner J. (2018). Impact of oxidative stress on telomere biology. Differentiation.

[11] Majerska J., Feretzaki M., Glousker G., Lingner J. (2018). Transformation-induced stress at telomeres is counteracted through changes in the telomeric proteome including SAMHD1. Life Sci. Alliance.

[12] Power J.H.T., Asad S., Chataway T., Chegini F., Manavis J., Temlett J.A., Jensen P.H., Blumbergs P.C., Gai W.-P. (2008). Peroxiredoxin 6 in human brain: Molecular forms, cellular distribution and association with Alzheimer’s disease pathology. Acta Neuropathol..

[13] Youssef P., Chami B., Lim J., Middleton T., Sutherland G.T., Witting P.K. (2018). Evidence supporting oxidative stress in a moderately affected area of the brain in Alzheimer’s disease. Sci. Rep..

[14] El Eter E., Al-Masri A. (2015). Peroxiredoxin isoforms are associated with cardiovascular risk factors in type 2 diabetes mellitus. Braz. J. Med. Biol. Res..

[15] Stancill J.S., Broniowska K.A., Oleson B.J., Naatz A., Corbett J.A. (2019). Pancreatic beta-cells detoxify H_2_O_2_ through the peroxiredoxin/thioredoxin antioxidant system. J. Biol. Chem..

[16] Collins J.A., Wood S.T., Nelson K.J., Rowe M.A., Carlson C.S., Chubinskaya S., Poole L., Furdui C.M., Loeser R.F. (2016). Oxidative stress promotes peroxiredoxin hyperoxidation and attenuates pro-survival signaling in aging chondrocytes. J. Biol. Chem..

[17] Gertz M., Fischer F., Leipelt M., Wolters D., Steegborn C. (2009). Identification of Peroxiredoxin 1 as a novel interaction partner for the lifespan regulator protein p66Shc. Aging.

[18] Radyuk S., Michalak K., Klichko V.I., Benes J., Rebrin I., Sohal R.S., Orr W.C. (2009). Peroxiredoxin 5 confers protection against oxidative stress and apoptosis and also promotes longevity in Drosophila. Biochem. J..

[19] Zhang Y.-G., Wang L., Kaifu T., Li J., Li X., Li L. (2016). Featured Article: Accelerated decline of physical strength in peroxiredoxin-3 knockout mice. Exp. Biol. Med..

[20] Argyropoulou V., Goemaere J., Clippe A., Lefort C., Tissir F., Schakman O., Gailly P., Ahn M.-T., Guiot Y., Galant C. (2016). Peroxiredoxin-5 as a novel actor in inflammation and tumor suppression. Free Radic. Biol. Med..

[21] Egler R., Fernandes E., Rothermund K., Sereika S., De Souza-Pinto N., Jaruga P., Dizdaroglu M., Prochownik E.V. (2005). Regulation of reactive oxygen species, DNA damage and c-Myc function by peroxiredoxin 1. Oncogene.

[22] Neumann C.A., Krause D.S., Carman C.V., Das S., Dubey D.P., Abraham J.L., Bronson R.T., Fujiwara Y., Orkin S.H., Van Etten R.A. (2003). Essential role for the peroxiredoxin Prdx1 in erythrocyte antioxidant defence and tumour suppression. Nat. Cell Biol..

[23] Abbasi A., Corpeleijn E., Postmus D., Gansevoort R.T., de Jong P.E., Gans R., Struck J., Schulte J., Hillege H.L., van der Harst P. (2012). Peroxiredoxin 4, a novel circulating biomarker for oxidative stress and the risk of incident cardiovascular disease and all-cause mortality. J. Am. Hear. Assoc..

[24] Huang P.-C., Chiu C.-C., Chang H.-W., Wang Y.-S., Syue H.-H., Song Y.-C., Weng Z.-H., Tai M.-H., Wu C.-Y. (2017). Prdx1-encoded peroxiredoxin is important for vascular development in zebrafish. FEBS Lett..

[25] Nelson K., Messier T., Milczarek S., Saaman A., Beuschel S., Gandhi U., Heintz N., Smalley T., Lowther W.T., Cunniff B. (2021). Unique cellular and biochemical features of human mitochondrial peroxiredoxin 3 establish the molecular basis for its specific reaction with thiostrepton. Antioxidants.

[26] Poynton R.A., Peskin A., Haynes A.C., Lowther W.T., Hampton M.B., Winterbourn C.C. (2016). Kinetic analysis of structural influences on the susceptibility of peroxiredoxins 2 and 3 to hyperoxidation. Biochem. J..

[27] Pastor-Flores D., Talwar D., Pedre B., Dick T.P. (2020). Real-time monitoring of peroxiredoxin oligomerization dynamics in living cells. Proc. Natl. Acad. Sci. USA.

[28] Chae H.Z., Chung S.J., Rhee S.G. (1994). Thioredoxin-dependent peroxide reductase from yeast. J. Biol. Chem..

[29] Haynes A.C., Qian J., Reisz J.A., Furdui C.M., Lowther W.T. (2013). Molecular basis for the resistance of human mitochondrial 2-Cys peroxiredoxin 3 to hyperoxidation. J. Biol. Chem..

[30] Winterbourn C.C., Peskin A. (2016). Kinetic approaches to measuring peroxiredoxin reactivity. Mol. Cells.

[31] Forshaw T.E., Holmila R., Nelson K.J., Lewis J.E., Kemp M.L., Tsang A.W., Poole L.B., Lowther W.T., Furdui C.M. (2019). Peroxiredoxins in cancer and response to radiation therapies. Antioxidants.

[32] Bolduc J., Nelson K.J., Haynes A.C., Lee J., Reisz J.A., Graff A.H., Clodfelter J.E., Parsonage D., Poole L., Furdui C.M. (2018). Novel hyperoxidation resistance motifs in 2-Cys peroxiredoxins. J. Biol. Chem..

[33] Portillo-Ledesma S., Randall L.M., Parsonage D., Rizza J.D., Karplus P.A., Poole L., DeNicola A., Ferrer-Sueta G. (2018). Differential kinetics of two-cysteine peroxiredoxin disulfide formation reveal a novel model for peroxide sensing. Biochemistry.

[34] Cao Z., Roszak A.W., Gourlay L.J., Lindsay J.G., Isaacs N.W. (2005). Bovine mitochondrial peroxiredoxin III forms a two-ring catenane. Structure.

[35] Biteau B., Labarre J., Toledano M.B. (2003). ATP-dependent reduction of cysteine–sulphinic acid by *S. cerevisiae* sulphiredoxin. Nat. Cell Biol..

[36] Jönsson T.J., Johnson L.C., Lowther W.T. (2008). Structure of the sulphiredoxin–peroxiredoxin complex reveals an essential repair embrace. Nat. Cell Biol..

[37] Jönsson T.J., Johnson L.C., Lowther W. (2009). Protein Engineering of the Quaternary Sulfiredoxin·peroxiredoxin enzyme-substrate complex reveals the molecular basis for cysteine sulfinic acid phosphorylation. J. Biol. Chem..

[38] Boukhenouna S., Mazon H., Branlant G., Jacob C., Toledano M.B., Rahuel-Clermont S. (2015). Evidence that glutathione and the glutathione system efficiently recycle 1-Cys sulfiredoxin in vivo. Antioxid. Redox Signal..

[39] Otwinowski Z., Minor Z. (1997). Processing of X-ray diffraction data collected in oscillation mode. Methods Enzymol..

[40] McCoy A.J. (2006). Solving structures of protein complexes by molecular replacement with Phaser. Acta Crystallogr. Sect. D Biol. Crystallogr..

[41] Schröder E., Littlechil J., Lebedev A., Errington N., Vagin A., Isupov M. (2000). Crystal structure of decameric 2-Cys peroxiredoxin from human erythrocytes at 1.7Å resolution. Structure.

[42] Murshudov G.N., Vagin A.A., Dodson E.J. (1997). Refinement of macromolecular structures by the maximum-likelihood method. Acta Crystallogr. Sect. D Biol. Crystallogr..

[43] Emsley P., Cowtan K.D. (2004). Coot: Model-building tools for molecular graphics. Acta Crystallogr. Sect. D Biol. Crystallogr..

[44] Chen V.B., Arendall W.B., Headd J.J., Keedy D.A., Immormino R.M., Kapral G.J., Murray L.W., Richardson J.S., Richardson D.C. (2010). MolProbity: All-atom structure validation for macromolecular crystallography. Acta Crystallogr. Sect. D Biol. Crystallogr..

[45] Laskowski R., MacArthur M.W., Moss D.S., Thornton J. (1993). PROCHECK: A program to check the stereochemical quality of protein structures. J. Appl. Crystallogr..

[46] Collaborative Computational Project (1994). The CCP4 suite: Programs for protein crystallography. Acta Crystallogr. Sect. D Biol. Crystallogr..

[47] Nelson K.J., Parsonage D. (2011). Measurement of peroxiredoxin activity. Curr. Protoc. Toxicol..

[48] Parsonage D., Youngblood D.S., Sarma G.N., Wood Z.A., Karplus P.A., Poole L.B. (2005). Analysis of the link between enzymatic activity and oligomeric state in AhpC, a bacterial peroxiredoxin. Biochemistry.

[49] Yewdall N.A., Venugopal H., Desfosses A., Abrishami V., Yosaatmadja Y., Hampton M.B., Gerrard J.A., Goldstone D.C., Mitra A.K., Radjainia M. (2016). Structures of human peroxiredoxin 3 suggest self-chaperoning assembly that maintains catalytic state. Structure.

[50] Chang T.-S., Jeong W., Woo H.A., Lee S.M., Park S., Rhee S.G. (2004). Characterization of mammalian sulfiredoxin and its reactivation of hyperoxidized peroxiredoxin through reduction of cysteine sulfinic acid in the active site to cysteine. J. Biol. Chem..

[51] Jeong W., Park S.J., Chang T.-S., Lee D.-Y., Rhee S.G. (2006). Molecular mechanism of the reduction of cysteine sulfinic acid of peroxiredoxin to cysteine by mammalian sulfiredoxin. J. Biol. Chem..

[52] Bayer S., Low F.M., Hampton M.B., Winterbourn C.C. (2016). Interactions between peroxiredoxin 2, hemichrome and the erythrocyte membrane. Free Radic. Res..

[53] Lim J.C., Choi H.-I., Park Y.S., Nam H.W., Woo H.A., Kwon K.-S., Kim Y.S., Rhee S.G., Kim K., Chae H.Z. (2008). Irreversible oxidation of the active-site cysteine of peroxiredoxin to cysteine sulfonic acid for enhanced molecular chaperone activity. J. Biol. Chem..

[54] Moon J.C., Hah Y.-S., Kim W.Y., Jung B.G., Jang H.H., Lee J.R., Kim S.Y., Lee Y.M., Jeon M.G., Kim C.W. (2005). Oxidative stress-dependent structural and functional switching of a human 2-Cys peroxiredoxin isotype II that enhances HeLa cell resistance to H_2_O_2_-induced Cell Death. J. Biol. Chem..

[55] Noichri Y., Palais G., Ruby V., D’Autreaux B., Delaunay-Moisan A., Nyström T., Molin M., Toledano M. (2015). In vivo parameters influencing 2-Cys Prx oligomerization: The role of enzyme sulfinylation. Redox Biol..

[56] Ogasawara Y., Ohminato T., Nakamura Y., Ishii K. (2012). Structural and functional analysis of native peroxiredoxin 2 in human red blood cells. Int. J. Biochem. Cell Biol..

[57] Angelucci F., Saccoccia F., Ardini M., Boumis G., Brunori M., Di Leandro L., Ippoliti R., Miele A.E., Natoli G., Scotti S. (2013). Switching between the alternative structures and functions of a 2-Cys peroxiredoxin, by site-directed mutagenesis. J. Mol. Biol..

[58] Noh Y.H., Baek J.Y., Jeong W., Rhee S.G., Chang T.-S. (2009). Sulfiredoxin translocation into mitochondria plays a crucial role in reducing hyperoxidized peroxiredoxin III. J. Biol. Chem..

[59] Rhee S.G., Kil I.S. (2016). Mitochondrial H_2_O_2_ signaling is controlled by the concerted action of peroxiredoxin III and sulfiredoxin: Linking mitochondrial function to circadian rhythm. Free Radic. Biol. Med..

[60] Akter S., Fu L., Jung Y., Conte M.L., Lawson J.R., Lowther W.T., Sun R., Liu K., Yang J., Carroll K.S. (2018). Chemical proteomics reveals new targets of cysteine sulfinic acid reductase. Nat. Chem. Biol..

[61] Roussel X., Béchade G., Kriznik A., Van Dorsselaer A., Cianferani S., Branlant G., Rahuel-Clermont S. (2008). Evidence for the formation of a covalent thiosulfinate intermediate with peroxiredoxin in the catalytic mechanism of sulfiredoxin. J. Biol. Chem..

[62] Roussel X., Kriznik A., Richard C., Rahuel-Clermont S., Branlant G. (2009). Catalytic mechanism of sulfiredoxin from *Saccharomyces cerevisiae* passes through an oxidized disulfide sulfiredoxin intermediate that is reduced by thioredoxin. J. Biol. Chem..

[63] Módis K., Coletta C., Erdélyi K., Papapetropoulos A., Szabo C. (2013). Intramitochondrial hydrogen sulfide production by 3-mercaptopyruvate sulfurtransferase maintains mitochondrial electron flow and supports cellular bioenergetics. FASEB J..

[64] Teng H., Wu B., Zhao K., Yang G., Wu L., Wang R. (2013). Oxygen-sensitive mitochondrial accumulation of cystathionine beta-synthase mediated by Lon protease. Proc. Natl. Acad. Sci. USA.

[65] Benchoam D., Semelak J.A., Cuevasanta E., Mastrogiovanni M., Grassano J.S., Ferrer-Sueta G., Zeida A., Trujillo M., Möller M.N., Estrin D.A. (2020). Acidity and nucleophilic reactivity of glutathione persulfide. J. Biol. Chem..

