# Supplementary material for: Specificity of Human Sulfiredoxin for Reductant and Peroxiredoxin Oligomeric State
Source: Antioxidants (Basel). 2021 Jun 11;10(6):946. doi: 10.3390/antiox10060946 (PMC8230665; doi:10.3390/antiox10060946)
Supplement: Supplementary file 1 [file antioxidants-10-00946-s001.zip › antioxidants-1237380-supplementary.pdf]

SUPPLEMENTARY INFORMATION

Specificity of Human Sulfiredoxin for Reductant and Peroxiredoxin Oligomeric State

Thomas E. Forshaw[1], Julie A. Reisz[1], Kimberly J. Nelson[2], Rajesh Gumpena[2], J. Reed Lawson[2], Thomas J. Jönsson[2], Hanzhi Wu[1], Jill E. Clodfelter[2], Lynnette C. Johnson[2], Cristina M. Furdui[1,a,b,*], W. Todd Lowther[2,a,b,c*]

[1] Department of Internal Medicine, Section on Molecular Medicine, and [2] Department of Biochemistry, Wake Forest School of Medicine, Medical Center Boulevard, Winston-Salem, North Carolina 27157.

[a] Center for Redox Biology and Medicine, [b] Comprehensive Cancer Center, and [c] Center for Structural Biology, Wake Forest School of Medicine, Medical Center Boulevard, Winston-Salem, North Carolina 27157

*Co-corresponding authors

To whom correspondence should be addressed:

W. Todd Lowther, Center for Structural Biology and Department of Biochemistry, Wake Forest School of Medicine, Medical Center Blvd., Winston-Salem, NC 27157. Tel.: 336-716-7230; Fax: 336-713-1283; E-mail: tlowther@wakehealth.edu.

Cristina M. Furdui, Department of Internal Medicine, Section on Molecular Medicine, Wake Forest School of Medicine; Medical Center Blvd., Winston-Salem, NC 27157. Tel.: 336-716-2697; Fax: 336-716-1214; E-mail: cfurdui@wakehealth.edu.

**Table S1.** Crystallographic data and refinement statistics.	
	**Srx-Prx1 decameric complex**
**Data Collection**	
Wavelength (Å)	1.1
Resolution range (Å)	50.00-3.00 (3.05-3.00)
Space group	*C*2
Unit cell (Å, °)	330.8 109.9 260.1 90 122.3 90
Total reflections	865913
Unique reflections	160698 (8051)
Multiplicity	5.4 (4.3)
Completeness (%)	99.8 (99.6)
Mean I/σI	9.05 (1.16)
Wilson B-factor ($Å^2$)	52.2
R-merge	0.167 (0.993)
R-meas	0.185 (1.129)
R-pim	0.078 (0.526)
$CC^{1/2}$ (highest resolution shell)	0.661
CC* (highest resolution shell)	0.994
**Refinement**	
Reflections used in refinement	141655 (4416)
Reflections used for R-free	7475 (233)
R-work	0.192
R-free	0.230
Number of non-hydrogen atoms	44942
macromolecules	44487
ligands	255
solvent	200
RMS (bonds)	0.008
RMS (angles)	1.484
Ramachandran favored (%)	90.3
Ramachandran allowed (%)	8.9
Generously allowed (%)	0.7
Disallowed (%)	0.1
Clashscore	3.74
Average B-factor ($Å^2$)	
macromolecules	67.45
ligands	95.85
solvent	38.88
Crystallization conditions	100 mM citric acid pH 4.5<br>100 mM CsCl<br>26% PEG 400
PDB code	7LJ1

Table S2. k_{SOH} formation rates as determined by HRP competition assay.

	Rate ($\times 10^6$ $M^{-1}s^{-1}$)
Prx2	27.8 ± 7.3
Prx2-E	8.5 ± 1.7
Prx3	17.0 ± 3.9
Prx3-EE	15.2 ± 4.1

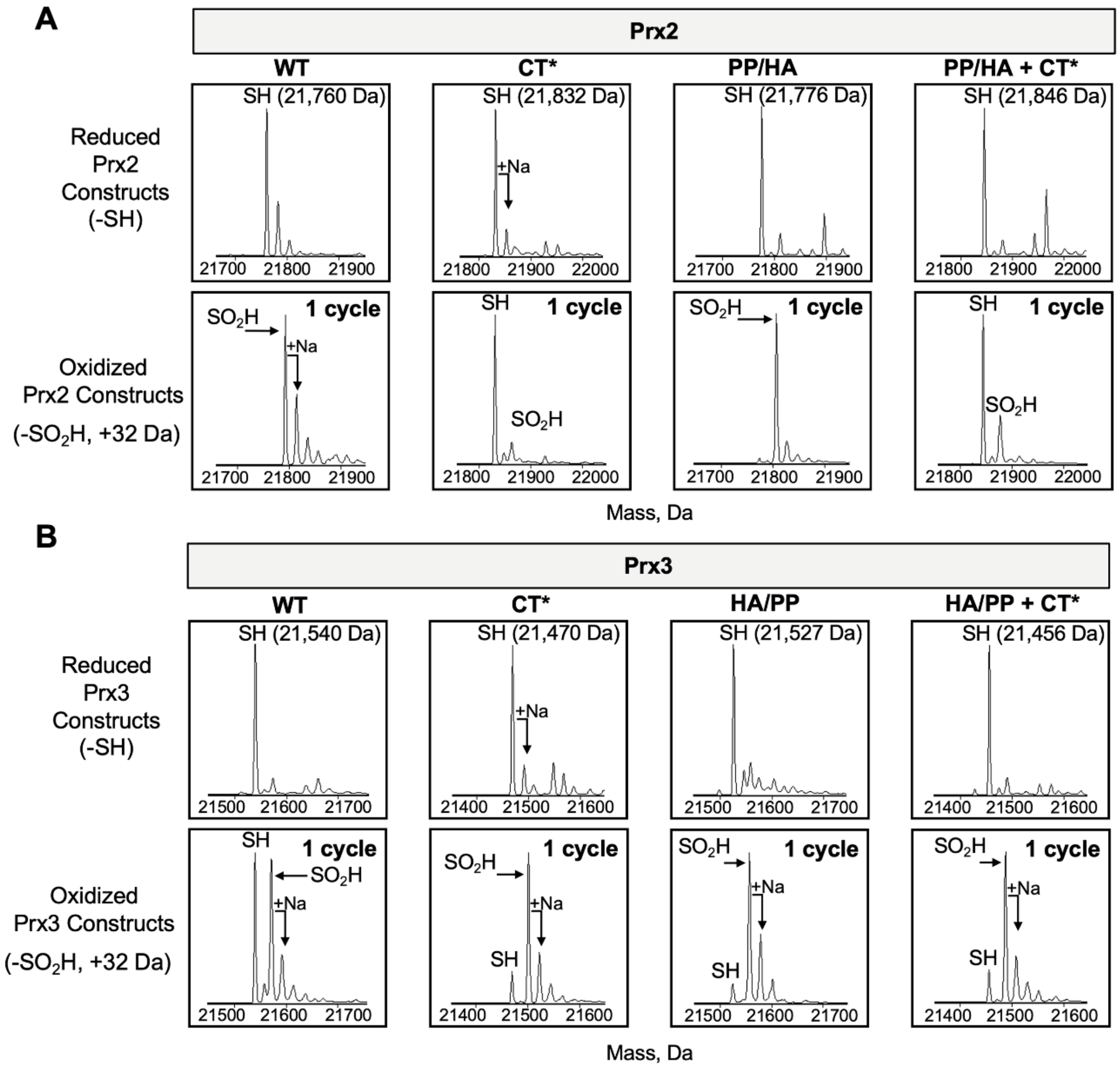

Figure S1. Hyperoxidation of Prx2 and Prx3 chimeras monitored by ESI-TOF MS. Hyperoxidation of Prx2 and Prx3 proteins was carried out by addition of H_2O_2 (all Prx2 WT and mutants 0.2 mM; all Prx3 WT and mutants 2.0 mM) every 30 minutes (one cycle) to Prx (~1 mg/mL) in the presence of DTT (50 mM). *A*, ESI-TOF MS spectra of WT Prx2 and chimeras prior to H_2O_2 addition (top) and subsequent 30 min redox cycle to induce hyperoxidation (bottom). *B*, ESI-TOF MS spectra of WT Prx3 and chimeras prior to H_2O_2 addition (top) and subsequent 30 min redox cycle to induce hyperoxidation (bottom).
